# Variable inhibition of different *Legionella* species by antagonistic bacteria

**DOI:** 10.1128/aem.01164-25

**Published:** 2025-09-08

**Authors:** Alessio Cavallaro, Silke Probst, Tobias Duft, Max Rieder, Oliver Abo El Fateh, Josch Stricker, Marco Gabrielli, Serina Robinson, Frederik Hammes

**Affiliations:** 1 Department of Environmental Microbiology, Eawag, Swiss Federal Institute of Aquatic Science and Technology 28499, Dübendorf, Switzerland; 2 Department of Environmental Systems Science, Institute of Biogeochemistry and Pollutant Dynamics, ETH Zurich 587558, Zürich, Switzerland; Universidad de los Andes, Bogotá, Colombia

**Keywords:** *Legionella*, competition, antagonism, *Pseudomonas*, biosurfactants, viscosin, inhibition

## Abstract

The genus *Legionella* includes opportunistic pathogens inhabiting engineered aquatic ecosystems, where managing their presence and abundance is crucial for public health. In these environments, *Legionella* interact positively or negatively with multiple members of the microbial communities. Here, we identified bacteria and compounds with *Legionella*-antagonistic properties. We isolated 212 bacterial colonies from various water sources in Switzerland and screened them for their ability to inhibit one reference strain of *Legionella pneumophila*. Ten selected antagonistic isolates were subsequently tested with spot-on-lawn assays for inhibition toward seven environmental and two clinical isolates of *Legionella*, representing different species and strains. The antagonists produced highly variable inhibition patterns, highlighting distinct differences in susceptibility among *Legionella* species and even strains. Only three isolates, all identified as *Pseudomonas lurida*, inhibited all *Legionella* species. We analyzed the genomes of the antagonistic bacteria and identified genes for several probable inhibitory compounds. We specifically found the gene cluster for the biosurfactant viscosin to be uniquely encoded by all three *P. lurida* isolates. This compound was subsequently detected in the supernatant of co-cultures inoculated with *P. lurida* and selected *Legionella* strains. However, additional experiments with a viscosin-deficient mutant of *Pseudomonas fluorescens* did not definitively demonstrate the importance of this compound for *Legionella* inhibition. This study provides new insights on the ability of aquatic microorganisms to compete with *Legionella* in controlled laboratory settings. It specifically highlights the diversity across and within *Legionella* species in their resistance to external antagonistic stress and further supports the potential anti-*Legionella* activity of some biosurfactants. These results can contribute to the understanding of how different *Legionella* species inhabit separate niches in the environment and expand the discussion around alternative *Legionella* mitigation strategies.

**IMPORTANCE:**

Understanding the nature of bacterial interactions allows us to have a broader overview of their lifestyle and their differences, while also paving the way toward new research and potential applications. This paper provides insights into the response of multiple pathogenic species of *Legionella* to aquatic antagonistic bacteria, highlighting interesting intra-genus differences that are then discussed in light of potential ecological implications. Moreover, we identified new antagonistic bacteria toward *Legionella* spp. and identified the biosurfactant viscosin as one of the likely compounds active against at least some *Legionella* species. Together, these results suggest different properties and possibly different environmental behaviors within the genus *Legionella*, while adding to previous studies with newly identified inhibitory organisms and compounds. We furthermore discuss the possibility that the latter could be explored in future research in order to develop a biological-based mitigation strategy against *Legionella* in engineered aquatic ecosystems, based on strains and bioactive compounds identified in this and other studies.

## INTRODUCTION

Bacteria belonging to the genus *Legionella* are waterborne opportunistic pathogens and etiological agents of Legionnaires’ disease, one of the most reported water-associated illnesses and with increasing incidence worldwide (1–3). *Legionella pneumophila* accounts for more than 90% of these cases (1, 4), yet the broad *Legionella* genus comprises, to date, more than 70 species officially recognized (5), of which approximately half have already been described as human pathogens (6, 7). The ecology of these non-*pneumophila* species is often less studied, although relevant given the fact that many of them are co-occurring in the environment (8). *Legionella* is typically found in engineered aquatic ecosystems such as building plumbing or cooling towers (2, 9), where its presence is mostly managed through chemical and physical disinfection. However, *Legionella* often persists in the environment due to its physiology and ecological characteristics, such as the protection offered by biofilms and protists (3, 10). In fact, *Legionella* lives embedded in complex biofilms, where it interacts with multiple prokaryotic and eukaryotic organisms, creating specific environmental niches (10).

In recent years, several studies have investigated the action of various compounds of biological origin to inhibit the growth of *Legionella*. Many of these discoveries have been well summarized by Berjeaud and coworkers (11), who divided the identified compounds into five classes, including proteins (e.g., apolipophorin III and lactoferrin isolated from eukaryotic organisms), protein-derived peptides of synthetic origin, antimicrobial peptides (e.g., warnericin RK isolated from *Staphylococcus warneri*), essential oils, and biosurfactants (e.g., surfactin isolated from *Bacillus subtilis*). However, these were isolated in diverse biological contexts not specifically linked to *Legionella’s* natural habitats; hence, some of these mechanisms may not necessarily occur in natural environments.

Targeted competition experiments have explored the anti-*Legionella* potential of microorganisms isolated directly from *Legionella*’s natural aquatic habitats. For example, Guerrieri and coworkers (12) tested 80 bacteria isolated from tap water and found that 37 out of 80 were active against at least one of 26 strains of *L. pneumophila*. Similarly, Corre and coworkers (13) reported the antagonistic activity of 178 bacteria collected in their study toward one strain of *L. pneumophila*, while Paranjape and coworkers (14) described seven isolates from cooling towers being able to inhibit multiple strains of *L. pneumophila* and detected the presence of gene clusters potentially encoding for molecules potentially responsible for the inhibition. In most of these cases, bacteria belonging to the genus *Pseudomonas*, known to produce diverse secondary metabolites (15), were detected as the main antagonists.

While these competition studies provide valuable information on the identity and biological activity of several aquatic bacteria toward *Legionella*, the actual inhibitory mechanisms are rarely identified, with the exception of biosurfactants produced by *Pseudomonas* strains reported by Loiseau and coworkers (16). Moreover, the majority of antagonism studies were conducted using only *L. pneumophila* as the target strain, and therefore, little is known about how other *Legionella* species respond to antagonism.

In the present study, we expand on previous work by testing the inhibitory activity of multiple bacterial isolates obtained from different water sources in Switzerland through spot-on-lawn assays. Importantly, we specifically focused on how different *Legionella* species respond to the antagonistic activity. We moreover investigated the antagonistic isolates for the presence of potential biosynthetic gene clusters (BGCs) encoding specific secondary metabolites, as the probable cause for the inhibition, and then detected their presence through liquid chromatography and mass spectrometry (LC-MS). Finally, we discuss our results in a broader environmental perspective, highlighting the role of different *Legionella* species in establishing distinct ecological niches.

## MATERIALS AND METHODS

### Antagonist isolation

Water samples were collected from various locations and sources in Switzerland (Table S1) in 100 mL Schott bottles, and 1 mL of the same sample was subsequently plated onto Lysogeny Broth and R2A agar plates (200 µL per plate) that were incubated at 30°C for 5 days. Twelve colonies per sample were picked based on distinct morphological properties and re-plated for isolation. The isolates were subsequently cultured in liquid medium overnight, and glycerol stocks (25% [vol/vol]) were prepared for storage at −80°C.

### *Legionella* strains

A reference strain of *L. pneumophila* (DSM7513) was obtained from the German collection of microorganisms ( Deutsche Sammlung von Mikroorganismen und Zellkulturen [DSMZ]). The other *Legionella* isolates used in this study were obtained from the Swiss National *Legionella* Reference Center (CNRL) located in Bellinzona (CH). The strains were selected among confirmed human pathogens and originated from either environmental water samples or clinical samples. A full list of the provided strains can be found in Table 1.

**TABLE 1:** *Legionella* strains used in this study

Strain	Source	CNRL no.
*L. pneumophila* DSM7513	DSMZ strain collection	–^*a*^
*Legionella londiniensis*	Water	12160
*Legionella anisa*	Water	12157
*L. pneumophila* sg. 2-14	Water	12156
*L. pneumophila* sg. 1	Water	12158
*L. pneumophila* sg. 2-14	Water	12143
*Legionella feeleii*	Water	12087
*Legionella bozemanii*	Clinical	12061
*Legionella jordanis*	Water	12018
*Legionella longbeachae*	Clinical	12012

^*a*^ –, not applicable.

### Spot-on-lawn assay

**Fig 1:**
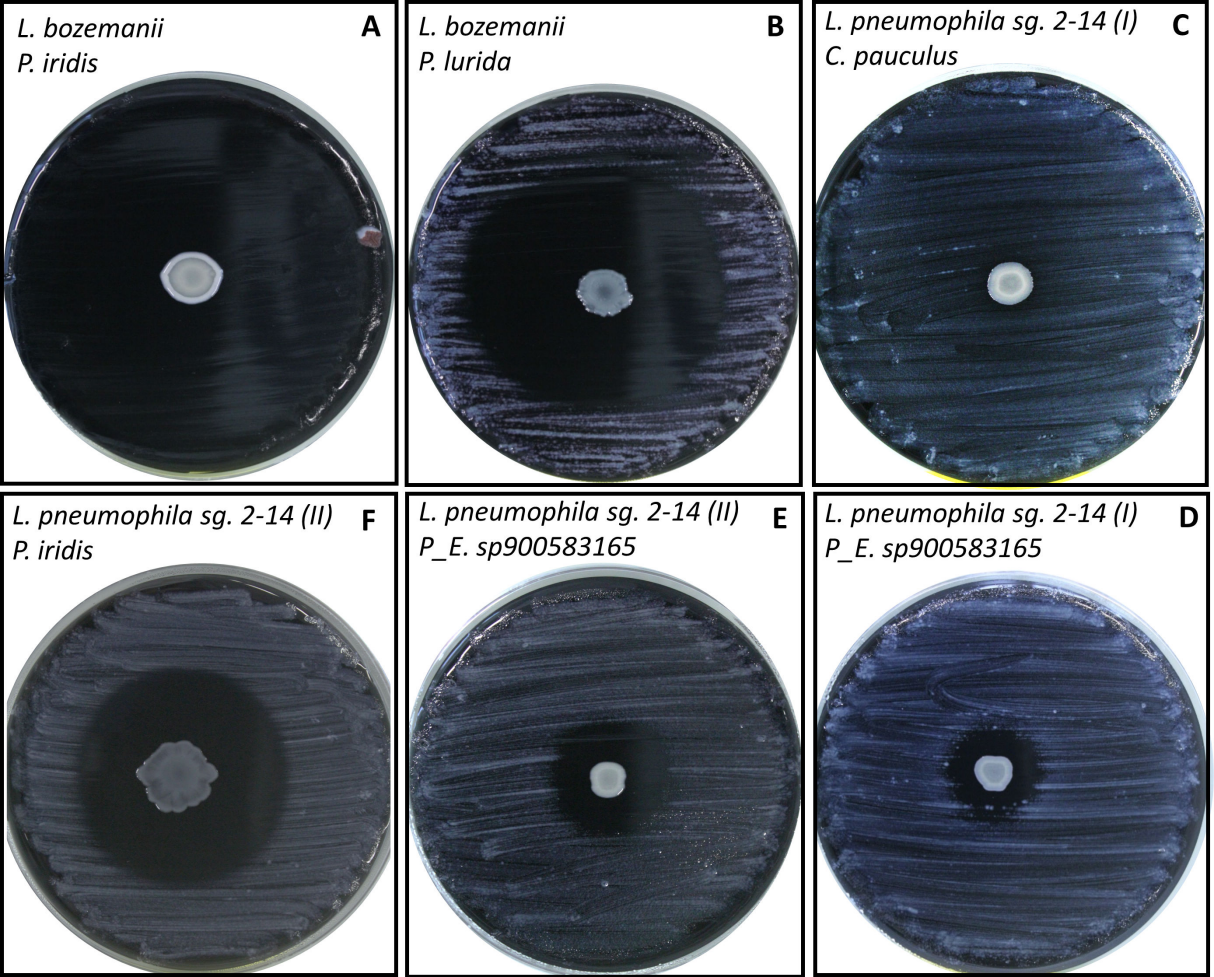
Six examples of spot-on-lawn experiments with multiple *Legionella* species and selected antagonistic isolates, showing variable visible outcomes. The top left corner provides the description of the *Legionella* strain used (top) and the antagonist (bottom). (**A**) Total inhibition of one *Legionella* species that is (**B**) only partially inhibited by a different antagonist. (**C**) No visible inhibition for a different *Legionella* strain, while (**D**) the same strain is mildly inhibited by a different antagonist. Different degrees of inhibition for the same *Legionella* species when subjected to the action of different antagonists in panels **E** and **F**.

Spot-on-lawn experiments were performed to evaluate the inhibition of *Legionella*. Briefly, *Legionella* strains were grown for 72 h on buffered charcoal yeast extract (BCYE) plates, and a colony was then transferred into Buffered Yeast Extract Broth (BYEB). After 72 h, the total cell count of *Legionella* in the culture was quantified using a CytoFLEX (Beckman Coulter, Brea, USA) flow cytometer in 250 µL aliquots stained using SYBR Green I (SG, Invitrogen AG, Basel, Switzerland; 10,000× diluted in Tris buffer, pH 8). Stained cells were incubated for 15 minutes at 37°C prior to analysis (17). After diluting the cultures to a concentration of 10^4^ cells/µL, 100 µL was inoculated on a BCYE agar plate and distributed with a cotton swab in order to form a dense lawn. To test the inhibitory activity of the bacterial isolates, fresh cultures were grown overnight and diluted to the same concentration as the *Legionella* strains. Ten microliters of the diluted cultures was spotted onto the agar plates previously inoculated with the *Legionella* lawn, and the plates were incubated at 30°C for 72 h. To test the inhibitory properties of the high-performance liquid chromatography (HPLC) fractions (see details in “Liquid-liquid extraction and HPLC fractionation,” below), these were diluted in methanol after being dried. Oxoid Blank Antimicrobial Susceptibility discs were soaked with 50 µL of each fraction and placed onto BCYE agar plates previously inoculated with the *Legionella* lawn (as above). An inhibitory activity was detected when a zone of non-visible growth was observed in the *Legionella* lawn around the colony or the susceptibility disc. As a further control to assess the potential contribution of the biosurfactant viscosin to the inhibition of the *Legionella* species, two strains of *Pseudomonas fluorescens* SBW25 (wild type [WT] and mutant for viscosin) were tested in spot-on-lawn assays as described above. These additional experiments were done without *Legionella anisa*. All the experiments were conducted in triplicate, and the plates were imaged using a camera set-up that allows for reproducible imaging. The inhibition was measured as the diameter of the inhibition zone, normalized by accounting for the colony size of the antagonist. The results are presented as the average diameter of the inhibition zone for triplicate experiments. The variation of inhibition in triplicate experiments is shown in Fig. S1. Examples of the spot-on-lawn assays conducted with bacterial isolates or discs are provided in Fig. 1 and Fig. S2.

### Whole-genome sequencing and bioinformatics

The genomic DNA of 10 selected antagonistic bacteria was isolated using the Qiagen DNeasy Blood and Tissue extraction kit. Whole-genome sequencing was performed in service by SeqCenter in Pittsburgh, PA (USA). Sample libraries were prepared using the Illumina DNA Prep kit and Integrated DNA Technologies (IDT) 10 bp unique dual indices (UDI) and sequenced on an Illumina NextSeq 2000, producing 2 × 151 bp reads. Demultiplexing, quality control, and adapter trimming were performed with bcl-convert (v3.9.3). Genomes were then assembled using the software SPAdes (v3.15.5) (18) and further quality-controlled for contamination and chimerism using the software CheckM2 (v1.0.2) (19) and GUNC (v1.0.6) (20). Taxonomy was assigned using GTDB-Tk (v2.3.2) through the function “classify_wf” (21). Genome mining for secondary metabolites was performed using the software antiSMASH (v7.0) (22) using the default parameters. The comparison between the two *Brevundimonas auriantiaca* strains was conducted using Anvio (v8) (23), using the pangenomic workflow through the program “anvi-pan-genome” (24). All plots and tables were generated in R (v4.3.1) and R Studio (v2023.06.0 + 421) using the packages ggplot2 (v3.4.2) and kable (v1.4.0).

### Liquid-liquid extraction and HPLC fractionation

Co-cultures of *Legionella jordanis* and *P. lurida* (I) were prepared by inoculating pre-grown bacteria diluted to a concentration of 10^4^ cells/µL, in a ratio of *Legionella:Pseudomonas* of 7:3. The co-culture was incubated for 72 h at 30°C in an orbital shaking incubator. After incubation, the culture was centrifuged using a Multifuge X Pro Series (ThermoScientific) at 10,000 g for 15 minutes, and the supernatant was separated from the pellet. The supernatant underwent liquid-liquid extraction with ethyl acetate. Briefly, 1.5 times the volume of ethyl acetate was added to the supernatant in a separation funnel, and the mixture was shaken thoroughly in order for the compounds to migrate to the organic phase. The extract was then transferred for subsequent analysis. The procedure was repeated three times using the same supernatant. The extracted organic phase was evaporated to dryness using a rotary evaporator and stored at −20°C until further use. The frozen extract obtained from the liquid-liquid extraction was reconstituted using 2 mL of methanol, and the precipitate was dissolved in a sonication bath. The sample was then centrifuged at 14,000 rpm for 1 minute in order to separate any particles from the liquid to be injected into the HPLC machine. The setup for the reverse phase HPLC consisted of an HPLC machine (Dionex UltiMate 300 Pump, ThermoScientific) and a column (C18[2], 250 × 21, Luna 5). The column was connected to a detector (Dionex UVD 340U Detector, Gynkotek) and a fraction collector (Model 2128, BIO-RAD). The collector switched between glass vials every minute, collecting approximately 10 mL of solution in each glass vial. The program for the liquid phase during the run was as follows: (i) increase of acetonitrile from 5% to 100% within 15 minutes; (ii) 15 minutes of 100% acetonitrile; (iii) decrease of acetonitrile from 100% to 5% within 5 minutes. Before starting the program, 1 mL of the 2 mL sample was added into the HPLC system using a syringe. A total of 31 fractions were collected. Two additional fractions were collected during the wash cycle, since an additional two peaks were detected. The fractions were then evaporated using a vacuum pump (Vacuum Pump V-600, Büchi). The concentrated fractions were dissolved in 1 mL of methanol and stored until further use.

### Liquid chromatography-mass spectrometry

The supernatant of the *L. jordanis–P. lurida* co-culture (above), along with supernatants from monocultures of *L. pneumophila* sg. 1, *L. jordanis*, and *P. lurida* (I and II), as well as the fractions that showed inhibition of *Legionella* in spot-on-lawn assays, was subjected to LC-MS to verify the presence of viscosin. All the supernatants underwent liquid-liquid extraction as described in “Liquid-liquid extraction and HPLC fractionation,” above. The samples were run through an HPLC system (Ultimate 3000, Dionex) connected to a column (2.6 µm XB-C18 150 × 4.6 mm, Phenomenex Kinetex). A mass spectrometer system (LTQ Orbitrap XL, Thermo ScientificTM) was coupled to the HPLC, resulting in HPLC-HRESI MS analytical analysis MS spectra. A specific program was used for each sample type using two solvents. Solvent A consisted of H_2_O and 0.1% formic acid, while solvent B consisted of acetonitrile and 0.1% formic acid. The injection volume of 10 µL was used for each sample with a flow rate of 0.7 mL/min. The solvent programs are shown in Table 2.

**TABLE 2:** Solvent programs used in LC-MS

Supernatant extract	Fractions
2 minutes with 5% of solvent B	2 minutes with 50% of solvent B
Increase of solvent B to 98% within 16 minutes	Increase of solvent B to 100% within 8 minutes
7 minutes with 98% of solvent B	10 minutes with 10% of solvent B
Decrease of solvent B to 5% within 0.1 minutes	Decrease of solvent B to 5% within 1 minute
	1 minute with 5% of solvent B

The same MS setup was used for all samples. The settings used can be found in Table S2. The spectra analysis was done using the software Xcalibur.

## RESULTS

### Isolation of the antagonists and inhibition of multiple *Legionella* species

A total of 212 isolates from 10 sources were initially screened for their ability to inhibit a reference strain of *L. pneumophila* DSM7513 using spot-on-lawn assays. In total, 34 isolates displayed some degree of inhibition through the formation of a clear zone in the *Legionella* lawn around the antagonist colony (see Fig. 1). From these, 10 isolates were selected, representing the broadest morphological diversity possible among the inhibitory bacteria. Analysis of the isolates’ genomes identified six isolates from the genus *Pseudomonas* (three isolates belonging to the species *P. lurida*, one *Pseudomonas iridis*, one *Pseudomonas lundensis,* and one *Pseudomonas_E sp900583165*). The rest of the isolates were classified as *B. auriantiaca* (two strains), *Cupriavidus pauculus* (one isolate), and *Sphingomonas* spp. (one isolate, no information available at the species level).

However, for two of these antagonistic isolates, we were unable to obtain pure cultures. Despite repeated attempts to isolate the species visibly growing together, the genomes assigned to *P. lurida* (III) and *P. lundensis* presented a high level of contamination (81.8% and 70.1%, respectively, analysis performed with CheckM2). An additional analysis performed by GUNC revealed that for *P. lurida* (III) 5,938 genes were assigned to the genus *Pseudomonas*, while 4,524 genes were attributed to *Chryseobacterium*; for *P. lundensis*, 4,205 genes were assigned to the genus *Pseudomonas*, and 4,814 genes were assigned to the genus *Bacillus*. Hence, the results from these two isolates should be considered in that context. The genomes of two *P. lurida* (I and II) isolates presented high similarity (average nucleotide identity [ANI] 100%; aligned fraction 99.94%). The third genome, because of the above-mentioned contamination, presented high similarity only in a portion of the genome (ANI 99.99%; aligned fraction 54.38). The two strains of *B. auriantiaca* presented an ANI of 99.98%, with an aligned fraction of 90.71%.

**Fig 2:**
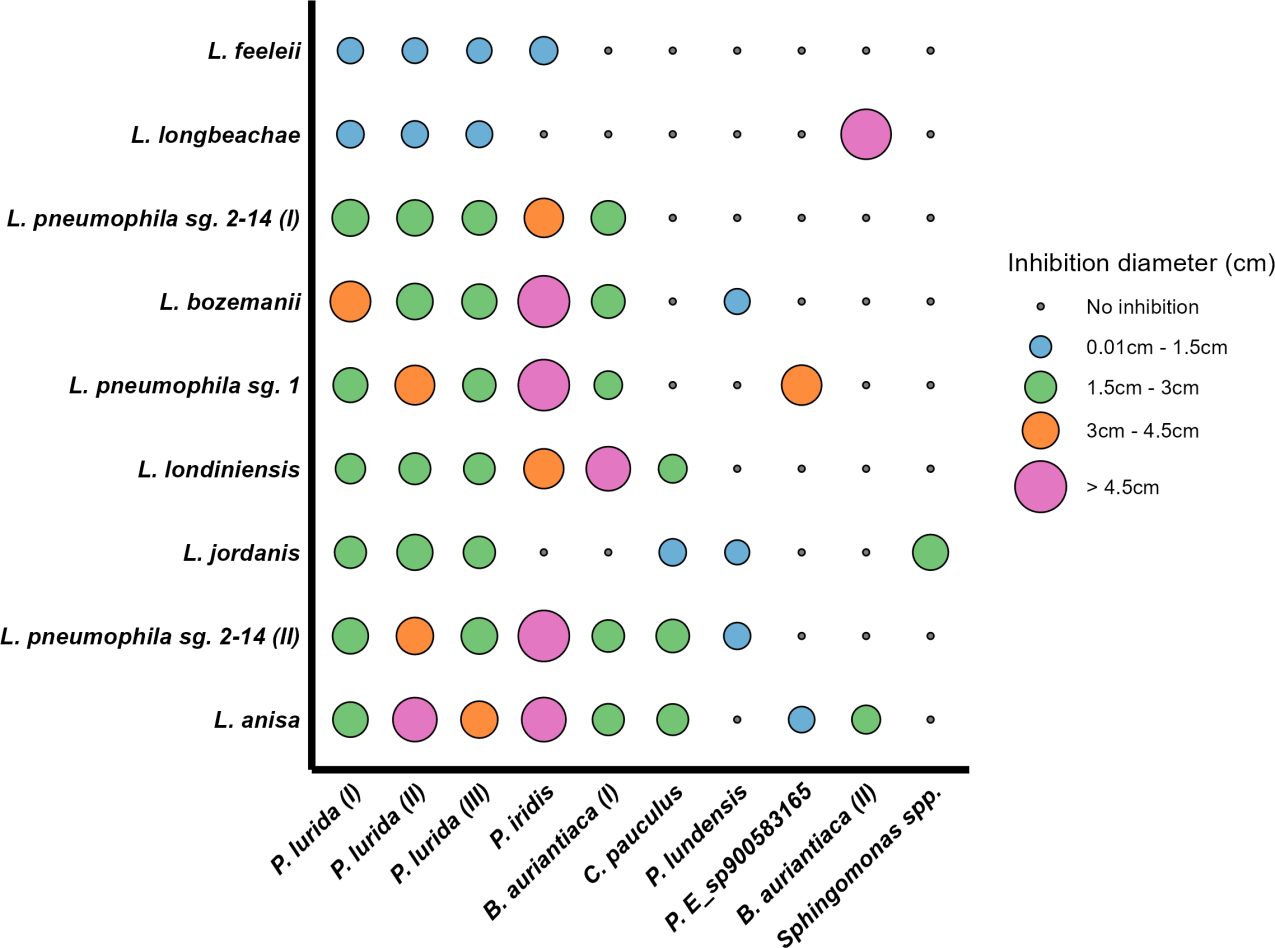
Results of the spot-on-lawn experiments in which the antagonistic isolates (displayed on the x-axis) were screened against nine clinical and environmental species of *Legionella* (displayed on the y-axis). A green circle indicates that an inhibition was observed on the plate, while a black point is displayed in the plot when no inhibition was detected. All the experiments were performed in triplicate; the size of the green circles is proportional to the degree of the inhibition measured as the average inhibition zone diameter, normalized for the colony size.

The selected isolates were then tested against multiple environmental and clinical isolates of *Legionella* (Fig. 1; Table 1). The three antagonists classified as *P. lurida* inhibited all the *Legionella* species tested, but the extent of the inhibition, measured as the size of the inhibition zone, differed, indicating that the antagonists exhibit possible strain-level variability in the production of antagonistic compounds (Fig. 2). *P. iridis* was unable to inhibit two *Legionella* species (*L. jordanis* and *Legionella longbeachae*). Moreover, in four cases, the inhibition caused by this antagonist was total, with no detectable *Legionella* growth on the entire plate, strongly suggesting that volatile compounds were involved in the inhibition. Even though two antagonists were both classified as *B. auriantiaca*, their inhibition profiles differed: in one case, six *Legionella* strains were inhibited with the diameter of the inhibition zone ranging from 1.53 cm to 4.64 cm; on the contrary, the second strain of *B. auriantiaca* only inhibited two *Legionella* species (*L. longbeachae* and *L. anisa*). Finally, *Pseudomonas_E sp900583165* and *Sphingomonas* spp. only inhibited two (*L. anisa* and *L. jordanis*) and one (*L. pneumophila* sg. 1) *Legionella* strains each.

With respect to the *Legionella* strains, *L. anisa* was the most susceptible strain, inhibited by eight different antagonists. Interestingly, the strains of *L. pneumophila* displayed a different inhibition pattern: while *L. pneumophila* sg. 2-14 (II) was susceptible to seven antagonists, *L. pneumophila* sg. 2-14 (I) was inhibited in five cases and with different degrees of inhibition. Moreover, the *L. pneumophila* sg. 1 used in this second screening was not susceptible to the activity of the antagonistic bacteria in four experiments, although all the antagonistic bacteria were originally selected for their ability to inhibit the growth of a reference strain of the same serogroup (*L. pneumophila* sg. 1 DSM7513). Finally, *L. longbeachae* and *Legionella feeleii* were the least susceptible strains, inhibited only by four antagonistic strains, of which three were the *P. lurida* isolates, while one was in one case *P. iridis* (*L. feeleii*) and in the other *B. auriantiaca* (II; *L. longbeachae*).

### Genome mining for potential inhibitory compounds

The genomes of our isolates were analyzed with the software antiSMASH and revealed a broad range of BGCs, of which non-ribosomal peptides (NRP) represented the main class. Table 3 reports the BGCs that showed high similarity (>60%) to previously known BGCs annotated in the antiSMASH database (25). The data show how the three isolates of *P. lurida* that inhibited all the *Legionella* strains possessed a BGC with high similarity to the cluster encoding for the biosurfactant viscosin. Although the percent of similarity reported was different (100%; 75%; 68%), in the latter two cases, the BGC was located at the edge of a contig, not allowing for the full coverage of the cluster, and the missing parts were detected on other contigs for the two organisms.

**TABLE 3:** BGCs detected in the genomes of the antagonistic strains using antiSMASH^*a*^

Strain	Most similar known cluster	Percent similarity	Type	
*P. lurida* (I)	Viscosin	75%	NRP	
*P. lurida* (II)	Viscosin	100%	NRP	
*P. lurida* (III)	Viscosin	68%	NRP	
	Flexirubin	77%	Polyketide	
	Rhizomide	100%	NRP	
	Icosalide	100%	NRP:lipopeptide	
*P. iridis*	Hydrogen cyanide	100%	Hydrogen cyanide	
*B. auriantiaca* (I)	–^*b*^	–	–	
*C. pauculus*	–	–	–	
*P. lundensis*	Hydrogen cyanide	100%	Hydrogen cyanide	
	Gamexpeptide	100%	NRP	
*Pseudomonas_E sp900583165*	Rhizomide	100%	NRP	
	Kolossin	100%	NRP	
*B. auriantiaca* (II)	–	–	–	
*Sphingomonas spp*.	Zeaxanthin	100%	Terpene	

^*a*^ The BGCs have been selected based on the similarity to previously annotated clusters (>60% similarity).

^*b*^ –, no biosynthetic gene clusters could be assigned to a specific strain using the analysis performed by antiSMASH.

Moreover, one strain of *P. lurida* (III) had three additional BGCs showing high similarity to flexirubin, rhizomide, and icosalide, respectively, which are to be attributed to the genome of the contaminant species. In fact, an analysis of the source microorganisms for these BGCs, conducted using the antiSMASH database, revealed their compatibility with the genus *Chryseobacterium*, which is the putative contaminant of the *P. lurida* (III) strain (see above).

The genome of *P. iridis* showed the presence of a BGC assigned to hydrogen cyanide, which is a volatile compound produced by some microorganisms with high toxicity toward other prokaryotic and eukaryotic organisms (26). While the production of a volatile compound would be able to explain the total inhibition observed in four cases with respect to *P. iridis*, it is notable that the same BGC was detected in the genome of *P. lundensis*, for which such total inhibition of *Legionella* was never observed. A possible explanation is that the BGC is only expressed by one strain, but no expression data are available for this work. The two strains of *B. auriantiaca*, as well as *C. pauculus*, did not reveal the presence of any high-similarity BGCs, highlighting the possibility of the inhibition being caused by a novel or orphan compound unassigned to any BGCs. The largely different inhibition pattern observed for *B. aurantiaca* (I) and (II) is explained by genetic differences between the two strains. In fact, a comparison between the two genomes revealed that *B. aurantiaca* (I) possesses 1,599 groups of genes that are not present in the genome of *B. aurantiaca* (II), which are most likely responsible for the differential inhibition. Of these groups of genes, 1,047 were annotated according to the Clusters of Orthologous Genes (COG) database (27, 28). While these results suggest substantial functional diversity of two strains belonging to the same species, it is difficult to establish which genes are specifically responsible for the increased inhibition. A complete list of the group of genes and their related functions is available in the Supplementary Information. BGCs for rhizomide and kolossin were detected in the genome of *Pseudomonas_E sp900583165*, while the cluster for zeaxanthin was present in the genome of *Sphingomonas* spp. While rhizomide is a cyclic-lipodepsipeptide (29), kolossin is a large non-ribosomal peptide, to our knowledge isolated only from the microorganism *Photorhabdus luminescens* (30). Zeaxanthin is a common carotenoid produced by plants and some microorganisms (31). Subsequent work in our study focused on viscosin-producing bacteria, as the cluster was observed in multiple strains able to inhibit all the *Legionella* species tested and refers to a clear, characterized compound.

### Identification of compounds belonging to the viscosin family in the cultures

In order to test whether the compounds causing the inhibition were secreted extracellularly, selected strains of *Legionella* (*L. pneumophila* sg. 1; *L. jordanis*) were co-cultured with two strains of *P. lurida* (I; II) in BYEB medium, and the supernatant was tested against the same *Legionella* strains. *L. jordanis* was inhibited by the supernatant of all the combinations prepared, while no inhibition of *L. pneumophila* sg. 1 was observed, probably due to lower concentrations of the antagonistic compounds in the supernatant.

Supernatants from co-cultures of *L. jordanis* and *P. lurida* (I), along with monocultures of *Legionella* and the antagonistic strain, were then subjected to liquid-liquid extraction followed by LC-MS analysis. This analysis identified masses [M + H]+ corresponding to viscosin (retention time [RT]: 19.33 minutes; 1,126.69 m/z), with variable relative abundances ranging from 2E6 to 1.19E7 across all samples. Additionally, masses corresponding to the masses of Massetolide E (RT: 18.84 minutes; 1,112.68 m/z; relative abundances 8.9E4–2.75E6) and Massetolide D (RT: 19.99 minutes; 1,140.71 m/z; relative abundances 1.2E4–1.75E5) were detected. Complete LC-MS data details are provided in Fig. S3 to S5. MS/MS analysis further confirmed the molecular structure of viscosin in all samples where it was detected, as shown in Fig. S3 to S5.

**Fig 3:**
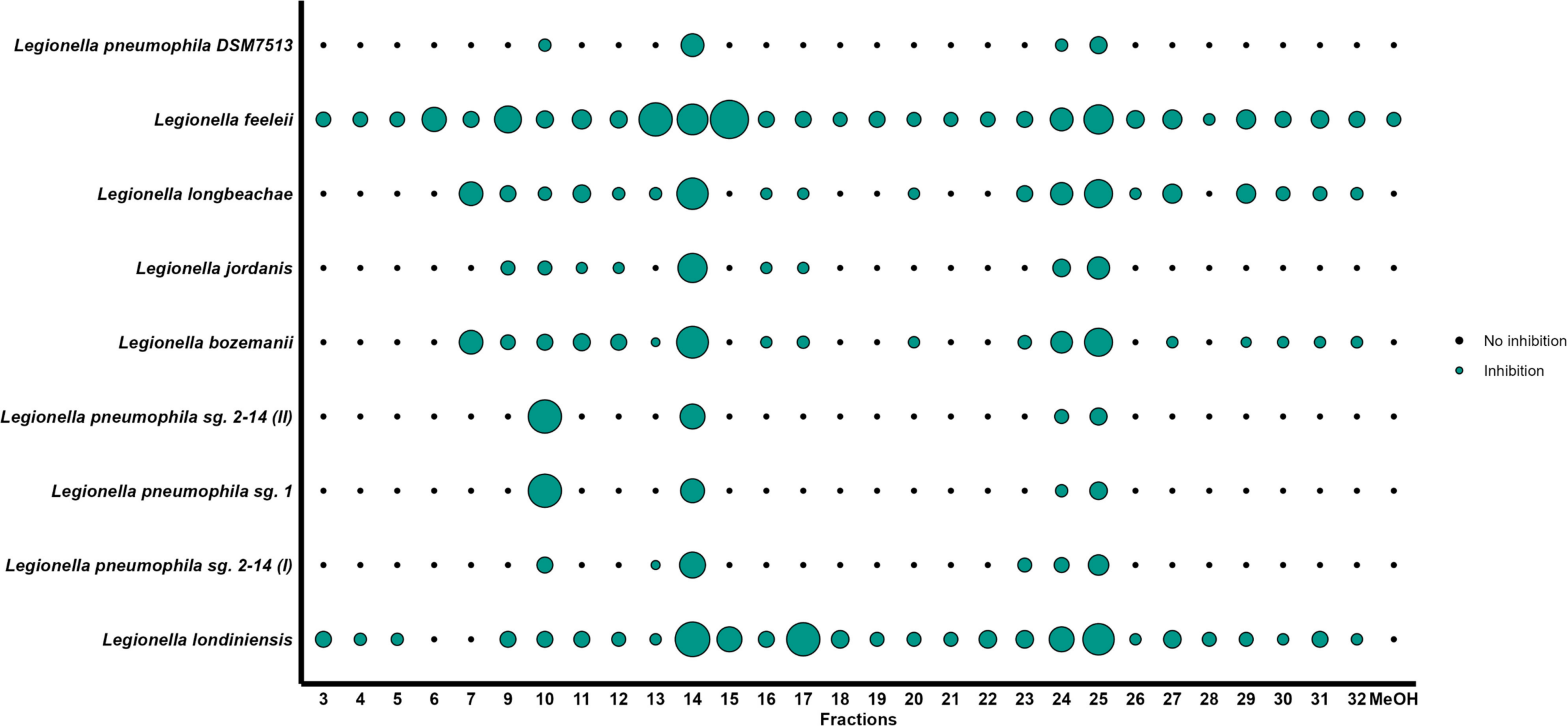
Results of the spot-on-lawn experiments in which the fractions obtained from the extracted supernatant, separated through HPLC (displayed in x-axis) were tested against the *Legionella* strains used in this work (displayed in y-axis). A green circle indicates that an inhibition was observed on the plate, while a black point is displayed when no inhibition was detected. All the experiments were performed in triplicate using blank antibiotic-susceptibility discs that were soaked with the respective fractions. The size of the green circles is proportional to the degree of the inhibition measured as the average inhibition zone diameter.

The supernatant of the co-culture *L. jordanis-P. lurida* (I) was afterward analyzed through preparative HPLC in order to separate the compounds and screen the fractions collected against all the *Legionella* species through spot-on-lawn assays. The results showed four fractions with antagonistic activity against all the *Legionella* strains (fractions 10, 14, 24, and 25; Fig. 3). However, given that the eluate was collected at 1-minute intervals rather than being synchronized with specific chromatographic peaks, it is conceivable that active compounds (and consequent inhibitory effects) were distributed across closely collected fractions.

LC-MS analysis of the inhibitory fractions identified the presence of masses [M + H]+ corresponding to viscosin in all four fractions (RT: 13 minutes; 1,126.69 m/z; relative abundances 2E6–1.1E7). Masses corresponding to Massetolide E were detected in fractions 10 (RT: 12.40 minutes; 1,112.68 m/z; relative abundance: 1.2E5) and fraction 25 (RT: 12.43 minutes; relative abundance: 5E3). Finally, a mass corresponding to the mass of Massetolide D was detected only in fraction 10 (RT: 12.63 minutes; 1,140.71 m/z; relative abundance: 4.5E3). MS/MS confirmed the molecular structure of viscosin (Fig. S6 to S8). Notably, masses which could not be assigned to specific compounds were detected in fractions 10 and 14 (RT: 10.77 minutes; 460.36 m/z, relative abundances: 1.1E8–1.6E8; RT: 13.23–13.35; 474.38 m/z; relative abundances: 1.7E7–1.35E8). An additional non-assigned mass was detected only in fraction 25 (RT: 7.66 minutes; 454 m/z; relative abundance 4.5E7). Additional experiments comparing a WT *P. fluorescens* strain with a viscosin-deficient mutant (Δ*ViscA*) (32) showed that the WT strain displayed stronger inhibition against only two (out of nine) *Legionella* species used in this study (*t*-test *P* value < 0.05; Fig. S9), supporting the potential role of viscosin as an inhibiting compound for at least some *Legionella* species. However, the significant residual inhibitory activity observed for the ΔviscA mutant (Fig. S9) indicates that other compounds also contribute to the overall anti-*Legionella* effect, at least in the case of *P. fluorescens*.

## DISCUSSION

This study aimed to isolate bacteria with antagonistic properties against *Legionella*. We isolated 10 bacteria (predominantly belonging to the genus *Pseudomonas*) with antagonistic activity toward one reference strain of *L. pneumophila* and observed variability in the inhibition pattern when we extended the screening to multiple *Legionella* species (Fig. 1 and 2). Furthermore, we investigated the genomes of the antagonists to identify potential inhibitory compounds (Table 3) and were able to fractionate and verify the antimicrobial activity of the supernatant of a co-culture *Legionella-P. lurida* (Fig. 3), identifying the biosurfactant viscosin as one of the likely *Legionella*-antagonistic compounds.

### Inhibition of *Legionella* spp. by aquatic bacteria

The study of microbial competition is an important tool for understanding how bacteria behave in stressful conditions, for the discovery of new antimicrobials, and for elucidating underlying ecological mechanisms driving the establishment of microorganisms in specific environments (33, 34). In recent years, increased attention has been given to the competitive interactions between aquatic bacteria and *Legionella*, possibly due to the necessity to gain deeper ecological insights on a problematic opportunistic pathogen and to investigate alternative mitigation strategies of this highly resistant genus (11, 35). Corre and coworkers (13) showed the antagonistic activity toward one strain of *L. pneumophila* by bacteria isolated from five different water sources, with a prevalence of antagonists belonging to the species *Pseudomonas* (*n* = 70) and *Aeromonas* (*n* = 19). Similar results were also presented by Guerrieri and coworkers (12), who showed the inhibition of several *L. pneumophila* strains by 80 aquatic bacteria, of which *Pseudomonas* represented the biggest fraction. Experiments performed using bacteria isolated from cooling towers described *Bacillus* spp., *Chryseobacterium* spp., *Cupriavidu*s spp., *Staphylococcus* spp., and *Stenotrophomonas* spp. as antagonists of nine *L. pneumophila* strains (14).

We purposefully built on these studies and isolated bacterial species antagonistic toward *Legionella* from different water sources in Switzerland. We detected bacteria belonging to the genus *Pseudomonas* as the main *Legionella* antagonists (Fig. 2). An isolation bias is present, as many other aquatic bacteria (and potential antagonists) cannot grow in standard media and temperatures used in this and previous studies (13). However, it is noticeable that previous molecular observations have also reported negative associations between *Pseudomonas* and *Legionella* in building plumbing systems (36, 37), cooling towers (38), and swimming pools (39). It is therefore possible that microorganisms belonging to these two genera share similar environmental preferences and occupy the same ecological niches. Specifically related to the genus *Pseudomonas*, we report for the first time the antagonistic activity of the species *P. lurida*, *P. iridis*, and *P. lundensis*, as well as *Legionella* inhibition by a novel *Pseudomonas*_E sp900583165. Combined with the data generated in other studies, this suggests that multiple *Pseudomonas* species can inhibit *Legionella*. Moreover, our experiments show anti-*Legionella* activity of bacteria belonging to the genus *Brevundimonas*, which have previously been associated with positive effects on the growth of *L. pneumophila* (40). These results suggest a variable outcome in the interactions between two genera, which can depend on specific species within the same genus (as our results also show in Fig. 2; discussed below) and on the general experimental and environmental conditions. Therefore, associations described by observational molecular studies, especially when the taxonomy is not resolved at the species level, might not reflect all the possible outcomes that interactions among members of different genera can have (41)

Overall, the results reported in this study provide additional new information about bacteria antagonistic to *Legionella*. However, the initial selection strategy for the antagonists was based on the inhibition of a reference strain of *L. pneumophila*. By doing this, given the variable inhibition reported in Fig. 2 (see “Variability in the inhibition patterns highlights *Legionella* diversity and potential ecological characteristics,” below), it is conceivable that we not only selected for more potential antagonists toward other *Legionella* species but also strains of *L. pneumophila*.

### Variability in the inhibition patterns highlights *Legionella* diversity and potential ecological characteristics

In the environment, bacteria compete for space and resources, using either interference or exploitative competition (42). The results of these competitive interactions are likely to have a strong influence on the composition of communities and how these are distributed in the environment (34, 43). We showed that *Legionella* species are inhibited by a range of antagonistic bacteria, and that the inhibition presented inter-species and intra-species variability (Fig. 2). This variability in the inhibition pattern can be analyzed from an ecological perspective, providing some useful insights. For instance, Russel and colleagues (44) demonstrated that bacteria are likely to exhibit antagonism toward microorganisms that present metabolic similarities, hence sharing nutritional preferences. In the context of our study, given the already observed negative relationship between *Pseudomonas* and *Legionella*, this would reinforce the possibility that the two genera share similar environmental niches. This would especially be relevant for the extracellular phase of the lifecycle of *Legionella*, given its preference for dense environments likely occupied by *Pseudomonas* such as biofilms (3). Moreover, this interpretation of the obtained results suggests the possibility that different *Legionella* species and strains live within distinct ecological niches in the environment. In a previously published study from our group (41), we have already observed a high genetic variability in the 16S amplicon sequence variants associated with the genus *Legionella*, as well as distinct patterns in the way the various *Legionella* amplicon sequence variants (ASVs) correlated among themselves and the rest of the community, which would once more support the idea that different ecological niches are occupied. This would explain co-occurrence within the same larger environment. These observations would be in agreement with multiple studies suggesting that differences between related groups of bacteria are driven by the way they differentiate in the environment (5, 45–47).

Substantial information exists regarding the antagonistic capabilities of *Pseudomonas*, which is a genus of bacteria known to harbor many different inhibition strategies (15, 48). In this context, it has already been demonstrated that *Pseudomonas* strains isolated from different environmental niches show variable antagonistic activity (49). Notably, even at the species level, significant differences in the outcome of competitive interactions are present, as shown by Lyng and Kovàcs (50) when assessing the interactions between *Pseudomonas* and *Bacillus*. The same authors described broad diversity in secondary metabolites responsible for antagonism across members of the same species, and even more of the same genus, hypothesizing that differences in inhibition might be driven by acquisition of genes through horizontal gene transfer. Microorganisms have also evolved multiple traits to defend against the attack of their neighbors (51), and this translates to the possibility that variable outcomes in competition are not only due to different compounds produced by the antagonists. For example, in our study, we observed that some antagonists could inhibit some *Legionella*, but not others (Fig. 2); at the same time, in multiple cases, one *Legionella* species is inhibited by several different antagonists. The first situation suggests that *Legionella* species can use various resistance mechanisms, while the multitude of compounds produced by the antagonists can be responsible for the second scenario.

Regardless of the exact reason behind the variable outcome of the competitive interactions between *Legionella* and the antagonists found in this and other studies (whether related to the environment or specific antagonistic/defense mechanisms), differences are present at the species and strain level for the genus *Legionella*. In a recent study (5), we conducted a genome-wide investigation of the family *Legionellaceae* and reported a high level of diversity related to the number of species, cellular components, and metabolism. While these data indicate different behaviors in the environment, to our knowledge, genus-level differences of the responses toward antagonistic mechanisms have not been explored in a broader ecological perspective for *Legionella,* although this is important because of the pathogenic nature of many members of the genus (4). Purposefully, the approach undertaken in this study aimed at exploring the response of multiple *Legionella* species to antagonistic bacteria, bringing the experiment beyond the use of *L. pneumophila* alone. This type of ecological information will support distinguishing between species and provide insights on pathogenicity, control strategies, and sampling/detection approaches.

A few studies have also started to predict broader ecological information on community assembly from pairwise inhibition building models (52) and dedicated laboratory experiments (53). While our study was not designed to allow such predictions to be extrapolated (specifically for the lack of quantitative growth data), collecting the necessary information in order to generate these predictions represents an important step for future experiments. As for all the studies that deal with competition, considerations about higher-order interactions (54) must be made since many of the ecological theories are built using pairwise interactions. Moreover, all these microbial interactions should be analyzed in the light of the intracellular life cycle that *Legionella* goes through in the environment inside protists (3), which can give a different meaning to these competition experiments.

### Role of biosurfactants in the inhibition of *Legionella*

Bacterial competition, and in particular interference competition, is often manifested through the secretion of several antibacterial compounds (55). A few studies investigated the molecules responsible for the inhibition. Faucher and coworkers showed that a strain of *Pseudomonas alcaliphila*, commonly found in cooling towers, was able to inhibit *Legionella* through the production of toxoflavin (56). Corre and coworkers identified the anti-*Legionella* volatile compound 1-undecene (57), while the inhibitory activity of the novel compound warnericin from *Staphylococcus warneri* was first described by Héchard and coworkers (58). In two subsequent studies, Loiseau and coworkers first showed that surfactin was able to inhibit multiple *Legionella* species, which presented a low minimum inhibitory concentration compared to other gram-positive and -negative bacteria (59) and then illustrated the strong activity of multiple biosurfactants (e.g., rhamnolipids and lipopeptide mixtures) produced by *Pseudomonas* strains (16).

Consistent with these findings, we detected BGCs related to the biosurfactants viscosin, rhizomide, and icosalide in the genomes of the antagonists tested (Table 2). Interestingly, the three strains of *P. lurida* that exhibited the broadest spectrum of inhibition toward the *Legionella* species tested (Fig. 2) were the only ones to possess the cluster for viscosin. The fact that we detected the masses corresponding to those of viscosin and massetolides A and D in the supernatant and in the fractions obtained through HPLC is therefore a strong indication of the fact that biosurfactants are indeed among the compounds responsible for the inhibition observed. Viscosin is a lipopeptide produced by several *Pseudomonas* spp. by a non-ribosomal peptide synthetase with demonstrated antimicrobial and antiviral activity (60) and is involved in the processes of biofilm formation and dispersal (61). Interestingly, the mode of action of several anti-*Legionella* compounds (including biosurfactants) is directed toward the bacterial membrane, suggesting a high susceptibility of *Legionella* to these molecules (11, 16). Moreover, while biosurfactants seem to be mostly active toward gram-positive bacteria (62), *Legionella* represents an exception in this sense, highlighting that the composition of their membrane may drive increased susceptibility.

However, our experiments suggest that the inhibition of *Legionella* was also caused by other compounds (alone or in combination with biosurfactants). Follow-up experiments with a viscosin-deficient mutant of *P. fluorescens* (Fig. S9) only showed viscosin-inhibition of three *Legionella* strains, while our genomic data suggest the presence of multiple BGCs (Table 3). Hence, several molecules might be responsible for the inhibition observed, especially in those cases of variable *Legionella* inhibition. While we did not search for additional compounds in this study, the opportunity exists to conduct more targeted experiments in order to identify more anti-*Legionella* molecules, expand the list that many above-mentioned studies have contributed, and ultimately test the biological activity in more realistic settings with dedicated laboratory or pilot-scale experiments.

Given the importance of reducing the levels of *Legionella* in engineered aquatic ecosystems, the use of new compounds will represent an alternative to chemicals (e.g., chlorine) whose efficacy against *Legionella* is lacking consistency. The adoption of new compounds with low toxicity might also help the acceptance of water disinfection in countries (e.g., Switzerland or the Netherlands) where this is less common. However, a few considerations are necessary in this regard. Compounds of biological origin, although in many cases less toxic than chemically synthesized ones, are only obtained in small amounts due to their production process (63) and are therefore currently not affordable by the water industries. Moreover, public acceptance for such alternatives is likely difficult to obtain, and these approaches would rather find application in systems working with water that will not directly enter in contact with the public (e.g., cooling towers and process water). Finally, these mitigation strategies would not be effective against *Legionella* when they are internalized in their eukaryotic hosts. Nevertheless, some of these compounds (as demonstrated for biosurfactants) can promote the disruption of the biofilms (61, 63), overcoming the challenges related to the intracellular *Legionella*.

### Conclusions

- Multiple bacteria were able to inhibit different species and strains of *Legionella*, but the interaction results were variable in both outcome and degree of inhibition, demonstrating the importance of considering *Legionella* species diversity in antagonism studies.
- Four species of *Pseudomonas* (namely *P. lurida*, *P. iridis*, *P. lundensis*, and *P. E_sp900583165*) were described as *Legionella* antagonists for the first time in this study.
- Variable inhibition of *Legionella* by other strains and compounds could explain important ecological differences among *Legionella* species in the environment. This is relevant given the occurrence of multiple pathogenic *Legionella* species in the environment. However, additional dedicated studies are necessary to reinforce this hypothesis.
- The biosurfactant viscosin was identified as one of the compounds potentially responsible for the broad inhibition of *Legionella*, supporting previous observations that biosurfactants are potentially interesting anti-*Legionella* compounds.

## Data Availability

DNA sequencing data are available via the NCBI Sequence Read Archive (SRA) under accession number PRJEB89887.

## SUPPLEMENTAL MATERIAL

Supplemental material aem.01164-25-s0001.docx Tables S1 and S2; Fig. S1 to S9.

Table S3 aem.01164-25-s0002.xlsx The group of genes present in *B. aurantiaca* (I) and absent in *B. aurantiaca* (II), possibly justifying the increased inhibition.

ASM does not own the copyrights to Supplemental Material that may be linked to, or accessed through, an article. The authors have granted ASM a non-exclusive, world-wide license to publish the Supplemental Material files. Please contact the corresponding author directly for reuse.
