# Supplementary material for: Variable inhibition of different Legionella species by antagonistic bacteria
Source: Appl Environ Microbiol. 2025 Sep 8;91(10):e01164-25. doi: 10.1128/aem.01164-25 (PMC12542786; doi:10.1128/aem.01164-25)
Supplement: Supplemental material — Tables S1 and S2; Fig. S1 to S9. [file aem.01164-25-s0001.docx]

Supplementary Information for

Variable inhibition of different *Legionella* species by antagonistic bacteria

Alessio Cavallaro^1,2^, Silke Probst^1^, Tobias Duft^1,2^, Max Rieder^2^, Oliver Abo El Fateh^2^, Josch Stricker^2^, Marco Gabrielli^1^, Serina Robinson^1^, Frederik Hammes^1^*

^1^ Department of Environmental Microbiology, Eawag, Swiss Federal Institute of Aquatic Science and Technology, 8600 Dübendorf, Switzerland

^2^ Department of Environmental Systems Science, Institute of Biogeochemistry and Pollutant Dynamics, ETH Zurich, 8092 Zurich, Switzerland

* Corresponding author:

Name: Frederik Hammes

Tel.: +41 58 765 5372

Email: frederik.hammes@eawag.ch

**Table S1.** List of the sources and relative location for the water samples used in the present study. Every water sample was plated in two different growth media (LB medium and R2A medium). 1 mL of each sample was split into five different plates (200 μL per plate). The table also reports the number of isolates that showed inhibitory activity towards a reference strain of L. pneumophila DSM7513.

| Source | Location | Nr. of inhibitory isolates |
| --- | --- | --- |
| Tap water | Zurich (CH) | 0 |
| Tap water | Basel (CH) | 0 |
| Tap water | Dübendorf (CH) | 2 |
| Shower water | Zurich (CH) | 4 |
| Shower water | Basel (CH) | 6 |
| Shower water | Dübendorf (CH) | 1 |
| Lake water | Zurich (CH) | 2 |
| Pond water | Dübendorf (CH) | 19 |
| Groundwater | Dübendorf (CH) | 0 |
| Bottled water | - | 0 |


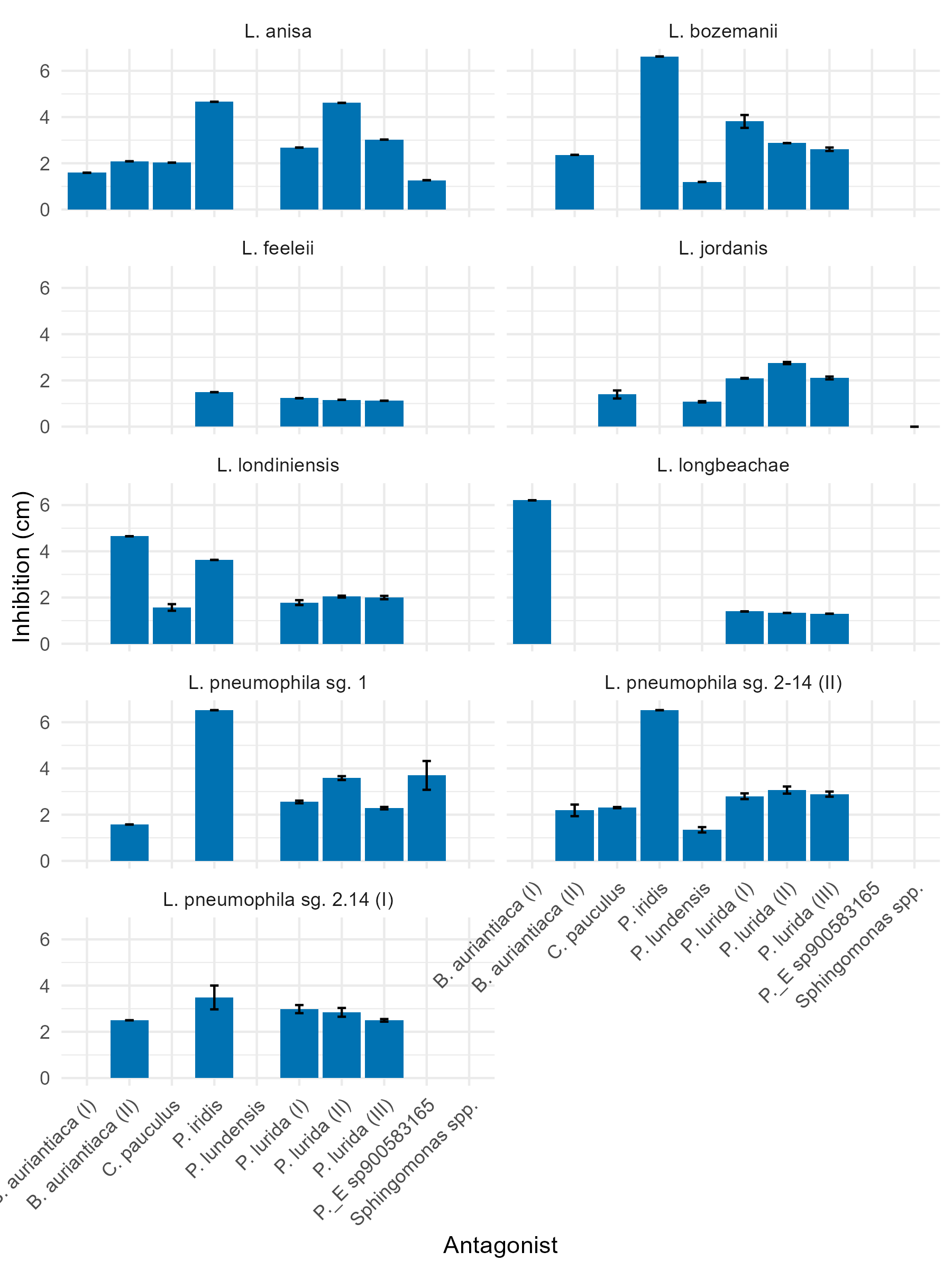


**Figure S1.** The figure shows a detailed overview of the inhibition of the Legionella species tested in this study. The x-axis reports the antagonistic strains, while the y-axis indicates the size of the inhibition (diameter of the inhibition zone normalized by the colony size, in cm). Each facets represent a different Legionella species, and the error bar shows the variability of inhibition across replicates.


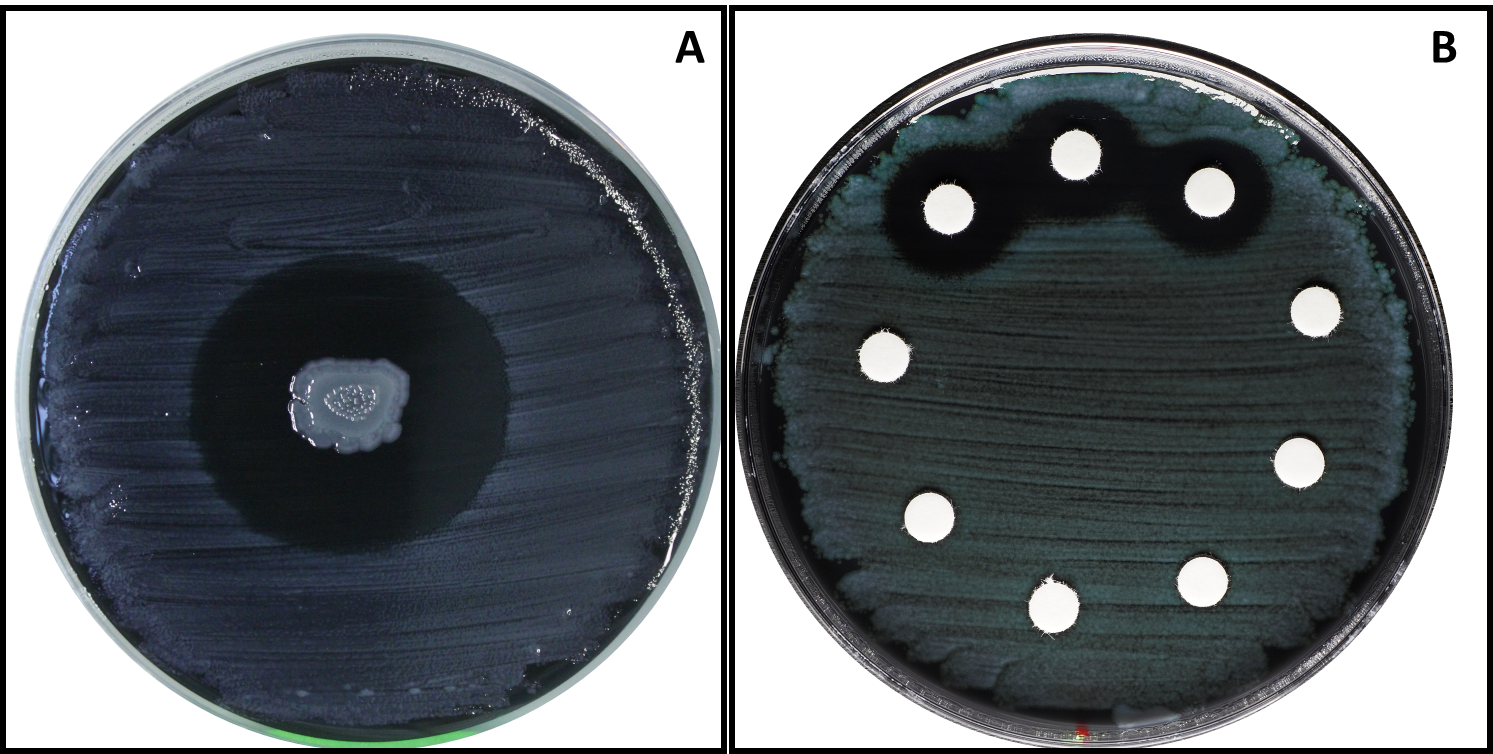


**Figure S2.** The figure shows examples of the two approaches used for the spot-on-lawn experiments performed in this study. A) An example of the spot-on-lawn conducted with the antagonistic bacteria. The antagonist (colony in the middle) is spotted on top of a lawn of Legionella; B) In order to test the fractions, blank antibiotic susceptibility discs were soaked with the solution of interest and, once dry, placed on a lawn of Legionella. In both cases, an inhibition is observed when Legionella does not grow around the colony/disc.

**Table S2.** Specific settings used for LC-MS analysis in all the experiments reported in this study. When applicable, values are given with their respective units.

| Setting | Value |
| --- | --- |
| Spray voltage | 3.5 kV |
| Capillary temperature | 320°C |
| Sheath gas | 57.5 |
| Aux gas | 16.25 |
| Spare gas | 3.25 |
| Probe heater | 462.50°C |
| Mode | Positive |
| Resolution | 30,000 |
| Microscans | 1 |
| Maximum IT | 100ms |
| Scan range | 150-200 m/z |


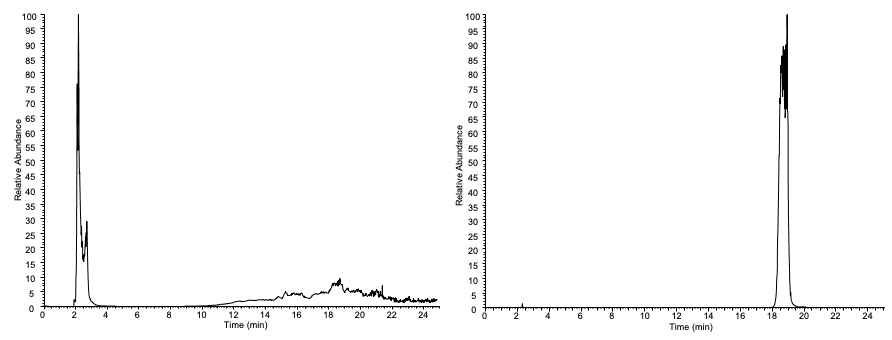


**A**

**B**

**C**


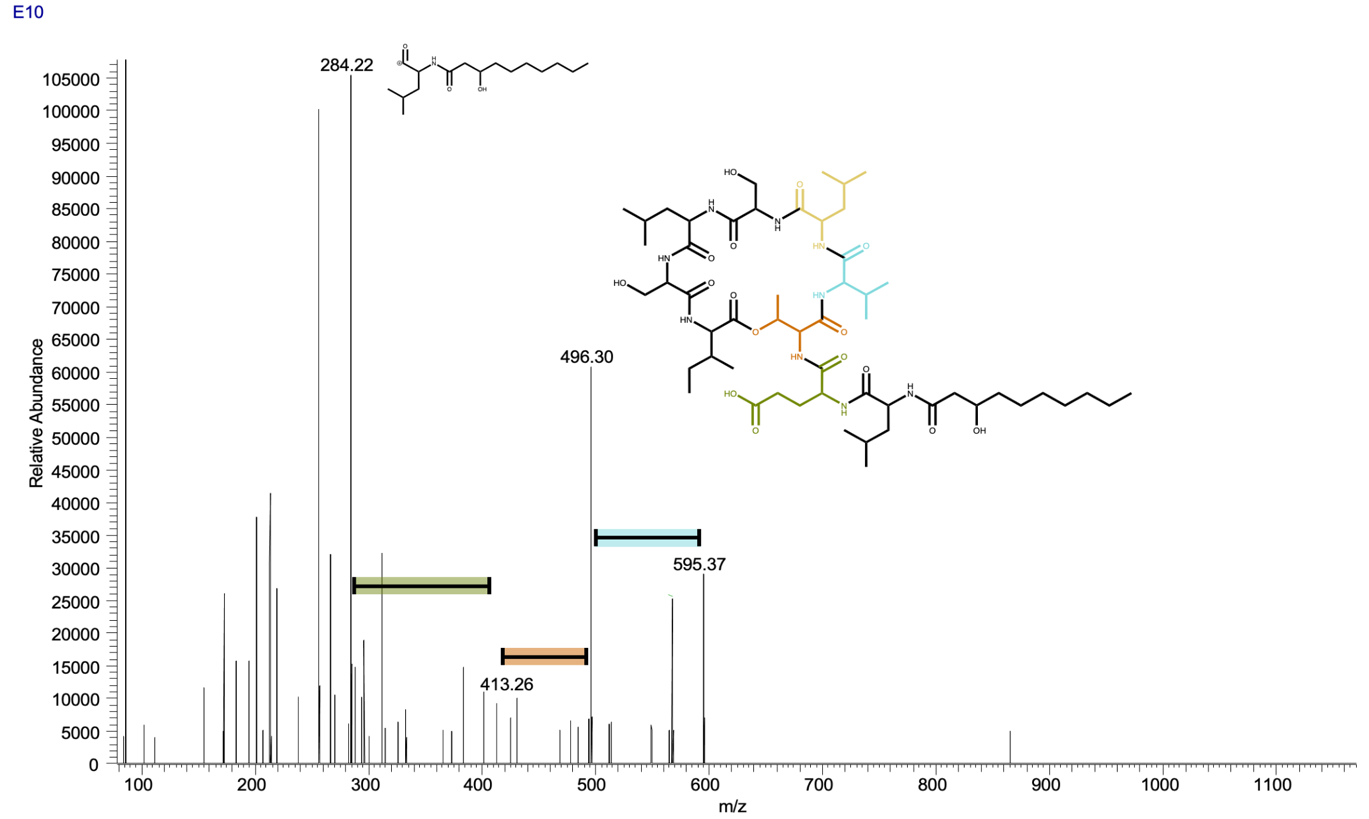


**Figure S3.** HR-LC-ESIMS data of the full *Pseudomonas lurida* (I) extract A) Total Ion Chromatogram (TIC) of the extracted culture. B) Extracted Ion Chromatogram (EIC) (m/z 1126.67 [M+H]) of viscosin. C) Measured fragments and ESI-MS/MS-spectrum of viscosin.


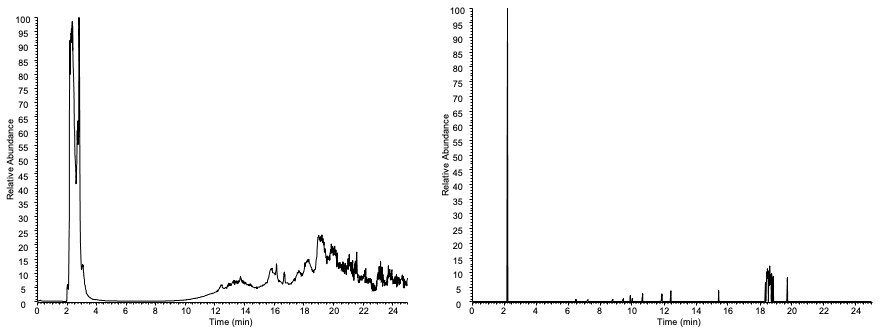


**B**

**A**

**Figure S4.** HR-LC-ESIMS data of the full *Legionella jordanis* extract A) Total Ion Chromatogram (TIC) of the extracted culture. B) Extracted Ion Chromatogram (EIC) (m/z 1126.67 [M+H]) of viscosin.

**B**

**A**


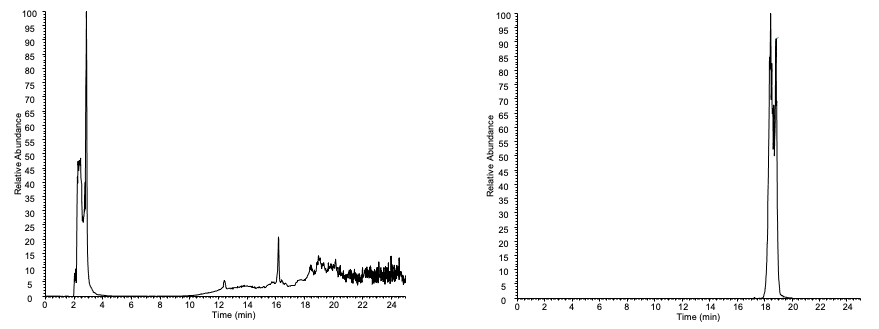


**C**


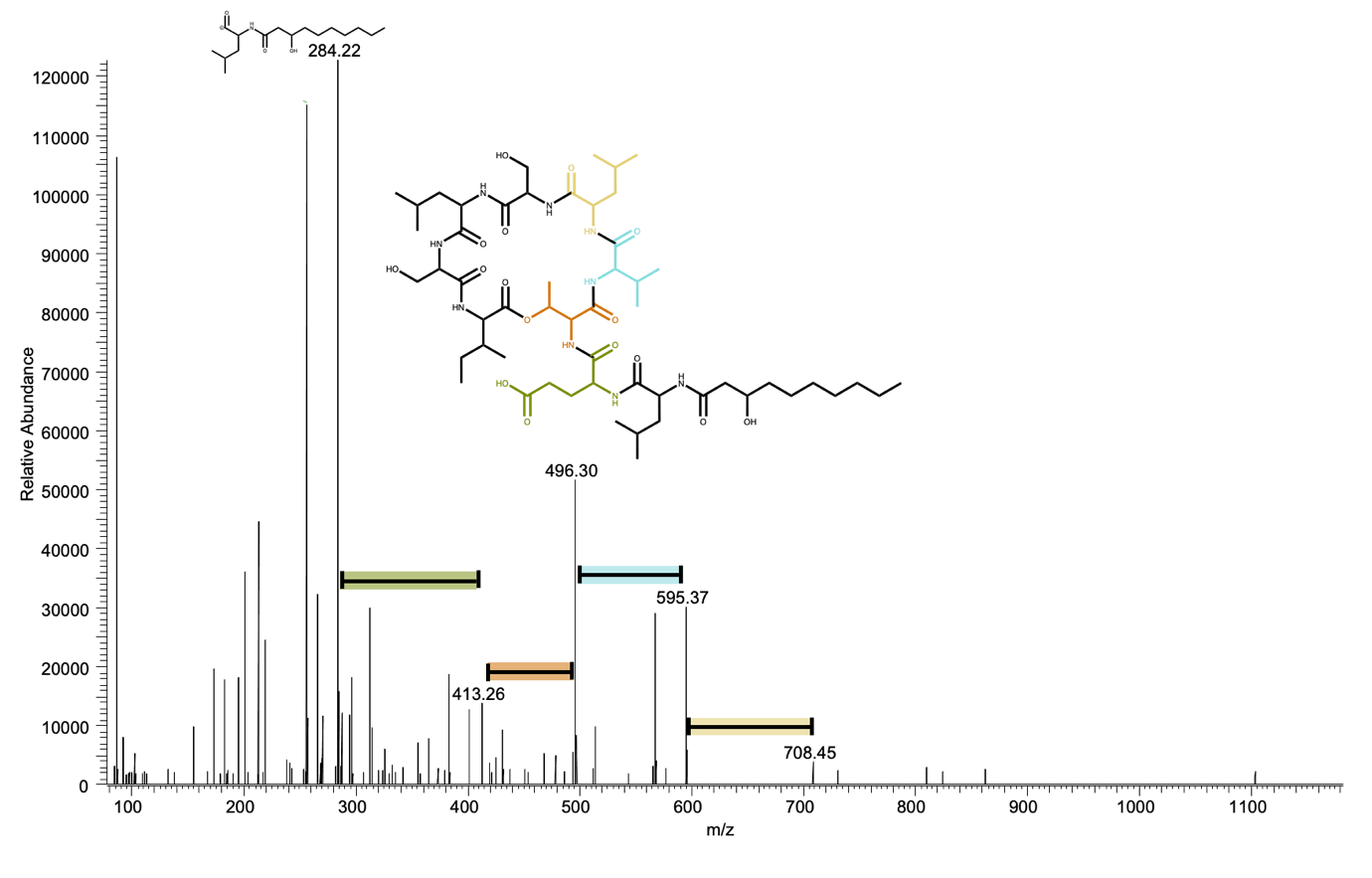


**Figure S5.** HR-LC-ESIMS data of the full *Pseudomonas* lurida (I) - *Legionella* jordanis co-culture extract A) Total Ion Chromatogram (TIC) of the extracted co-culture. B) Extracted Ion Chromatogram (EIC) (m/z 1126.67 [M+H]) of viscosin. C) Measured fragments and ESI-MS/MS-spectrum of viscosin.

**B**

**A**


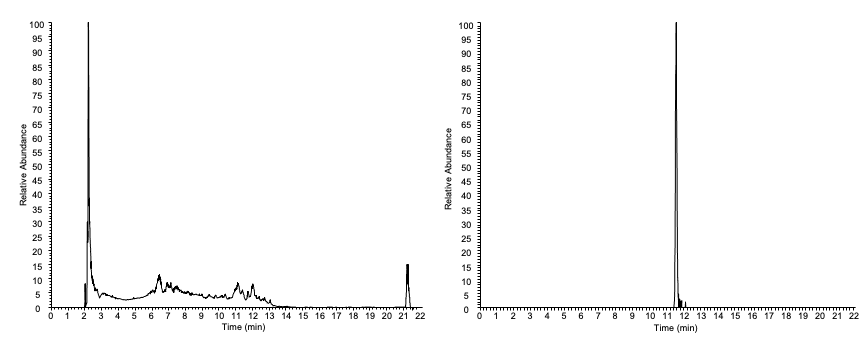


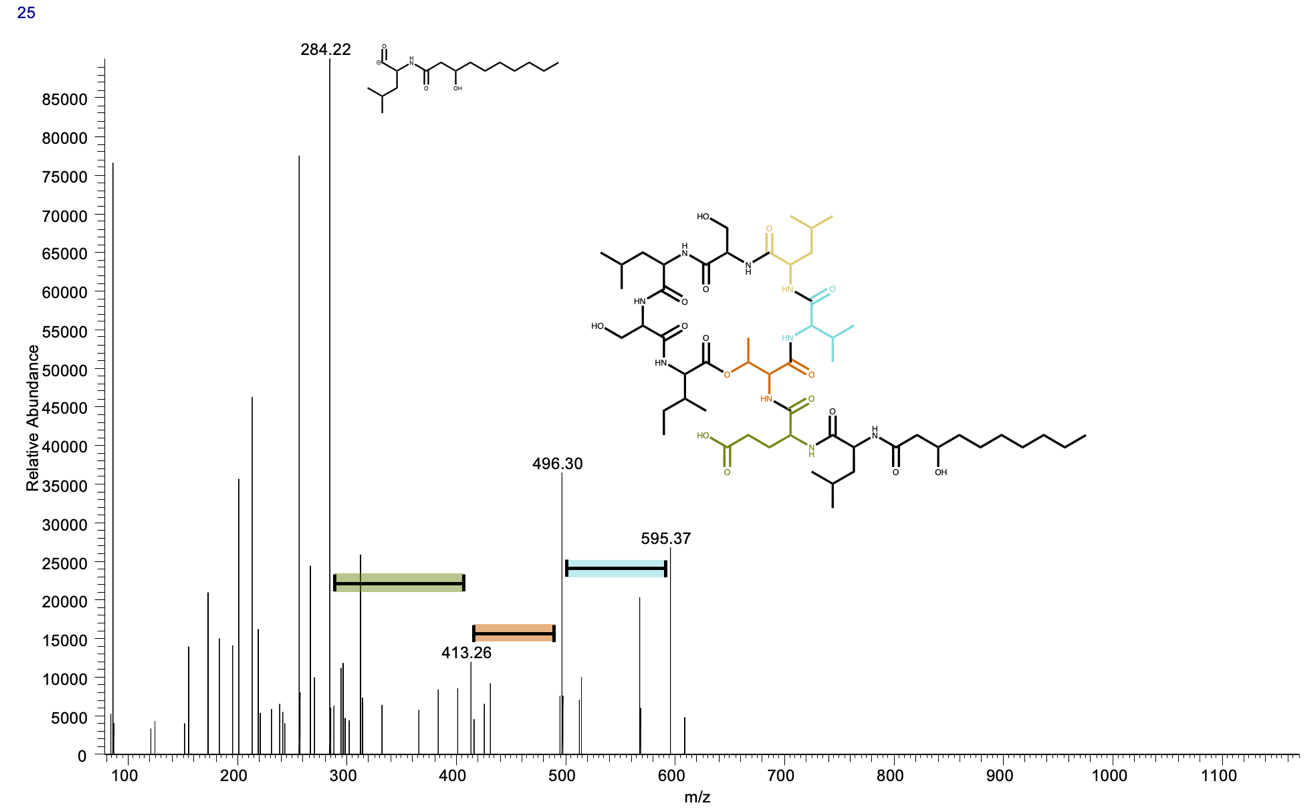


**C**

**Figure S6.** HR-LC-ESIMS data of the fraction 24 of the *Pseudomonas* lurida (I) -*Legionella* jordanis co-culture extract A) Total Ion Chromatogram (TIC) of the fraction 24 of the extracted co-culture (E10+18). B) Extracted Ion Chromatogram (EIC) (m/z 1126.67 [M+H]) of viscosin. C) Measured fragments and ESI-MS/MS-spectrum of viscosin.


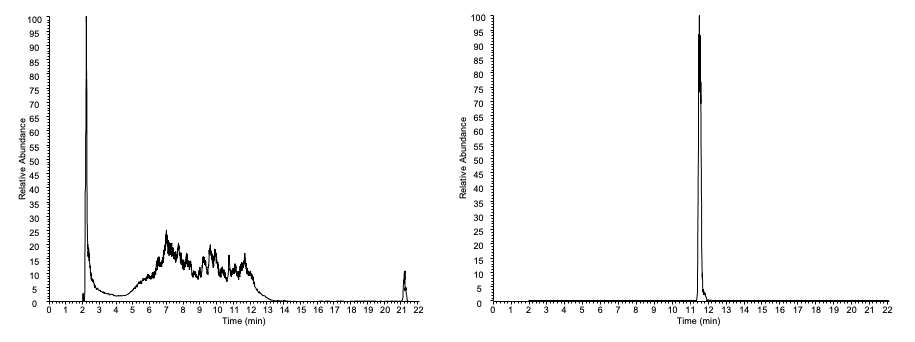


**B**

**A**

**C**


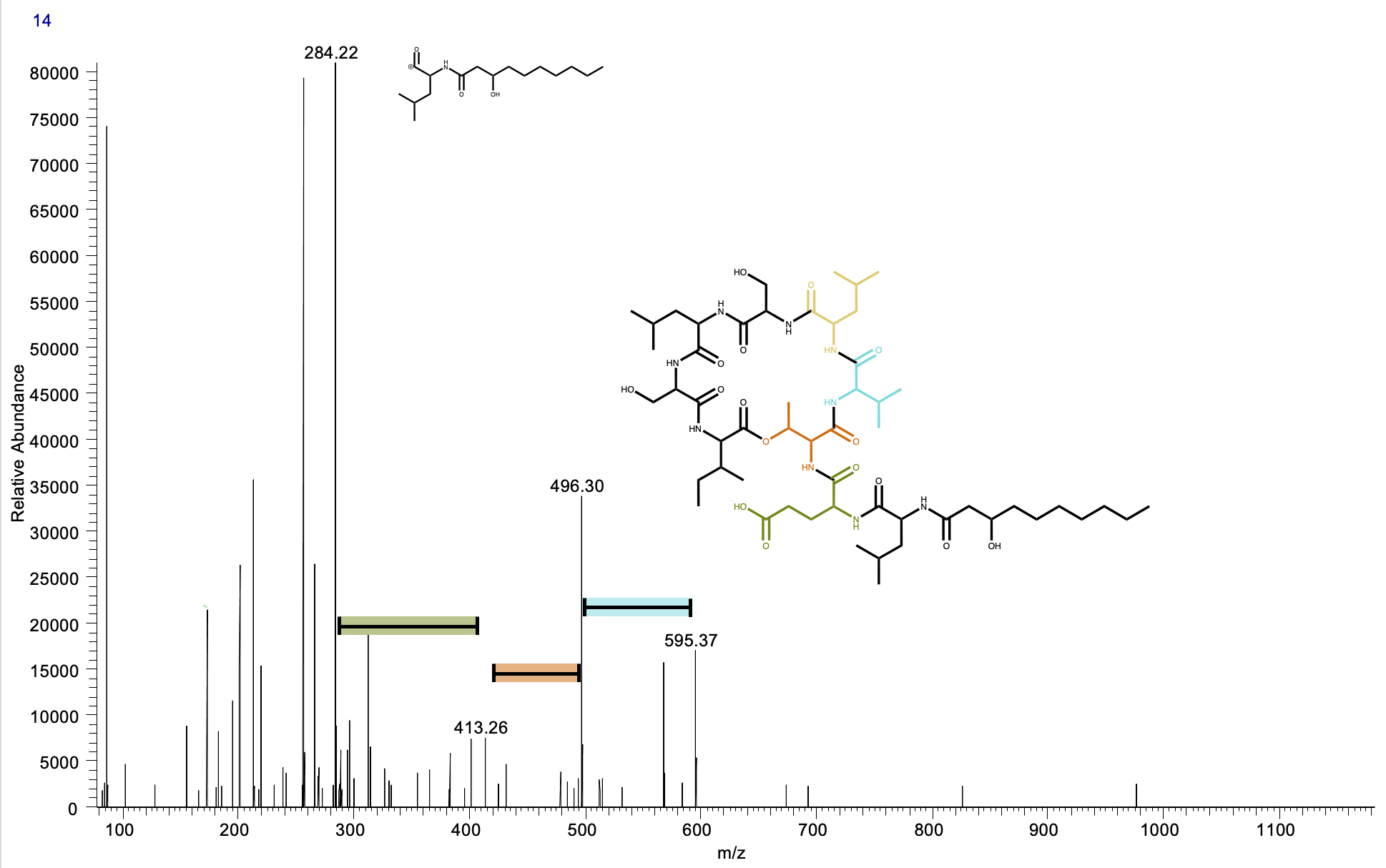


**Figure S7.** HR-LC-ESIMS data of the fraction 14 of the *Pseudomonas* *lurida* (I) - *Legionella* *jordanis* co-culture extract A) Total Ion Chromatogram (TIC) of the fraction 14 of the extracted co-culture. B) Extracted Ion Chromatogram (EIC) (m/z 1126.67 [M+H]) of viscosin. C) Measured fragments and ESI-MS/MS-spectrum of viscosin.

**B**

**A**


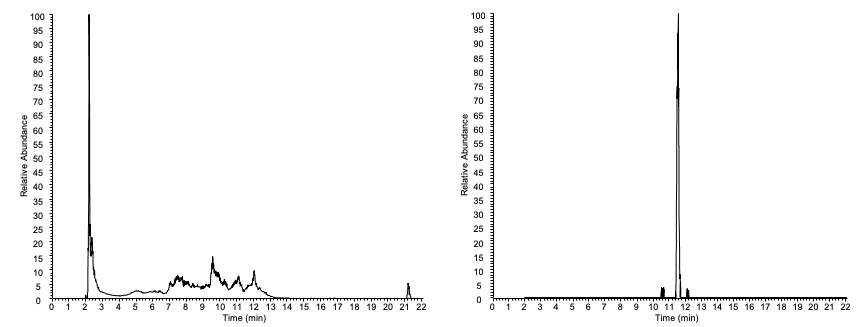


**C**


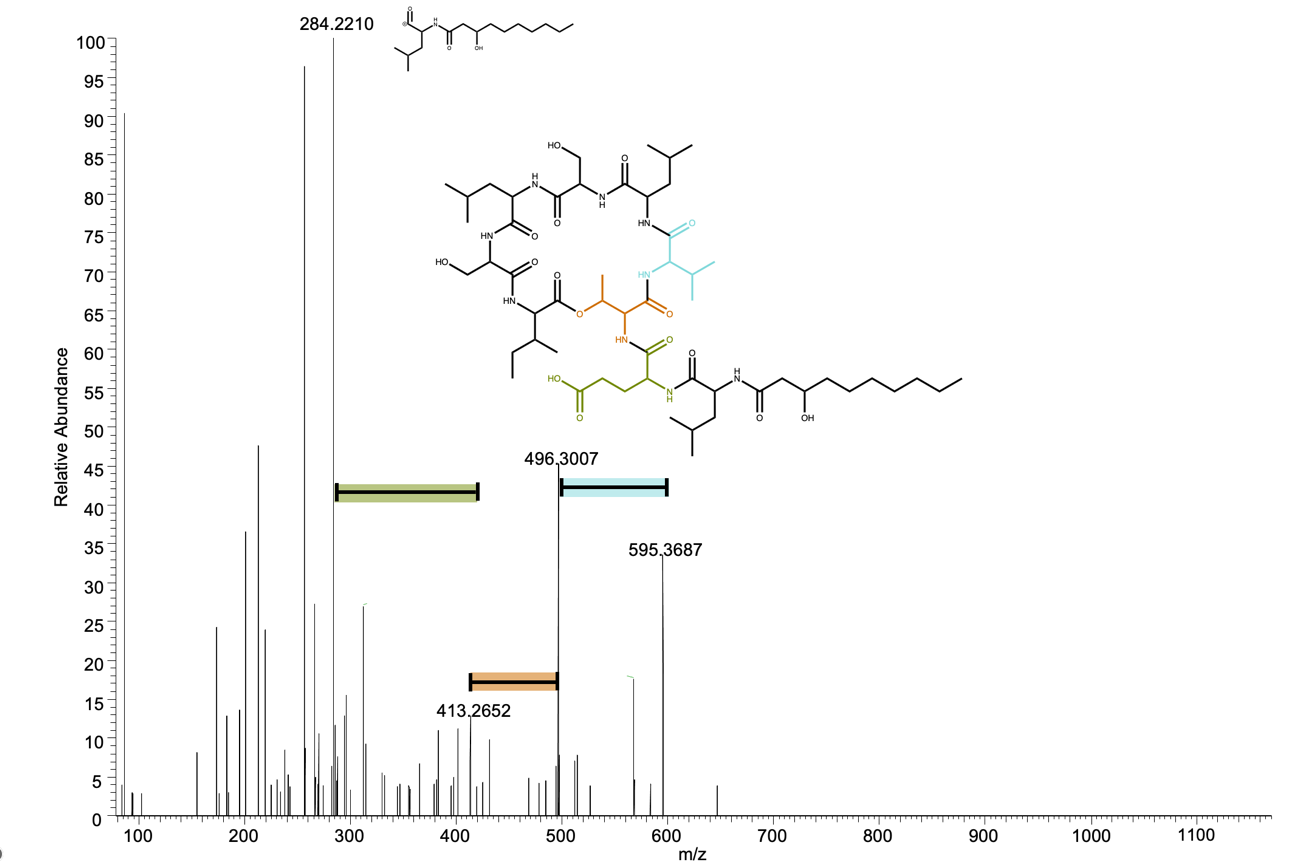


**Figure S8.** HR-LC-ESIMS data of the fraction 10 of the Pseudomonas *lurida –* *Legionella* *jordanis* co-culture extract A) Total Ion Chromatogram (TIC) of the fraction 10 of the extracted co-culture. B) Extracted Ion Chromatogram (EIC) (m/z 1126.67 [M+H]) of viscosin. C) Measured fragments and ESI-MS/MS-spectrum of viscosin.


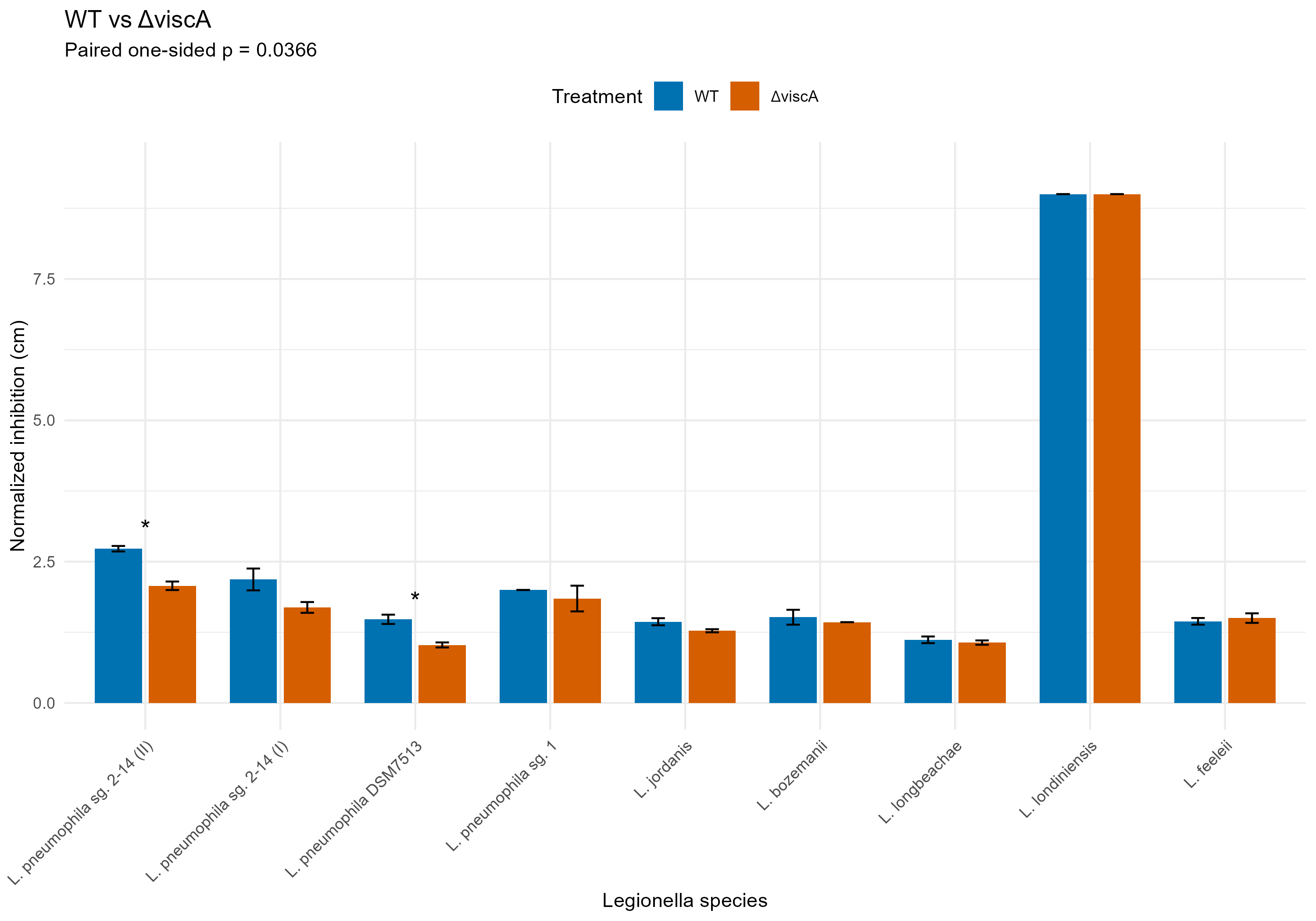


**Figure S9.** Grouped bar plot showing the inhibition caused by the WT viscosin producing strain (*P. fluorescens* SBW25) and the viscosin mutant strain (*P. fluorescens* SBW25 ΔviscA) towards the *Legionella* species tested in this study. The *Legionella* species are indicated on the x-axis, while the y-axis displays the inhibition expressed as the diameter of the inhibition zone normalized by the colony size (in cm). Statistical significance was assessed within each species by a paired one‐sided t-test (WT > ΔviscA), with p-values adjusted by the Benjamini–Hochberg FDR procedure; significance levels are indicated as * p < 0.05, ** p < 0.01, *** p < 0.001. A global paired one-sided t-test across all species gave p = 0.0366. For *L. longbeachae*, complete inhibition was observed in experiments conducted with both the WT and the mutant strain, while *L. anisa* failed to grow when this experiment was conducted. Although a global t-test suggests that the viscosin WT *P. fluorescens* inhibits *Legionella* significantly more (p-value <0.05), individual t-tests for each species show that only *L. pneumophila* sg. 2-14 (II) and *L. pneumophila* DSM7513 remained significant (p-value <0.05). Since it is not possible to determine whether the WT strain produces viscosin under the experimental conditions used here (BYEB; 30°C), nor to clarify the potential role of other inhibitory compounds produced by both WT and mutant strains, this experiment alone cannot conclusively determine if viscosin is the primary antagonistic molecule. However, previous experiments confirmed that viscosin is present in the supernatant of *Pseudomonas*–*Legionella* co-cultures, suggesting, together with the evidence generated by this experiment, that viscosin likely acts together with other secondary metabolites to produce the antagonistic activity observed here.
